# Primary mediastinal seminoma presenting with paraneoplastic anti-Hu encephalitis: a case report and literature review

**DOI:** 10.3389/fonc.2023.1156566

**Published:** 2023-09-15

**Authors:** Chelsey M. Williams, Derek B. Allison, Adam B. Coleman, Roshmita Bardhan, Jordan D. Miller, Zin W. Myint

**Affiliations:** ^1^ Department of Internal Medicine, University of Kentucky, Lexington, KY, United States; ^2^ Markey Cancer Center, University of Kentucky, Lexington, KY, United States; ^3^ Department of Pathology and Laboratory Medicine, University of Kentucky, Lexington, KY, United States; ^4^ Liberty University College of Osteopathic Medicine, Lynchburg, VA, United States; ^5^ University of Pikeville- Kentucky College of Osteopathic Medicine, Pikeville, KY, United States; ^6^ Division of Thoracic Surgery, University of Kentucky, Lexington, KY, United States; ^7^ Department of Internal Medicine, Division of Medical Oncology, University of Kentucky, Lexington, KY, United States

**Keywords:** primary mediastinal seminoma, anti-Hu, paraneoplastic encephalitis, ANNA-1, bilateral hearing loss, rhombencephalitis

## Abstract

Primary mediastinal seminomas are exceedingly rare tumors, often localized to the anterior mediastinum. They may present with numerous complications, including superior vena cava syndrome, chylothorax, and pericardial effusions. Less commonly, they may present with paraneoplastic encephalitis. In this report we describe a case of a 19-year-old male with no significant past medical history who presented with bilateral hearing loss, progressive neuropathy, and ataxia. Subsequently the patient was found to have mediastinal mass with a high-titer anti-Hu antibody. To our knowledge, only one other case of mediastinal seminoma presenting with anti-Hu antibodies has been described in the literature. In this report, we describe a rare case of mediastinal seminoma, describe treatment options, and discuss additional known cases presenting with paraneoplastic encephalitis.

## Introduction

First described in 1955 by Wolner et. al, primary mediastinal seminomas (PMS) are exceptionally rare germ cell tumors that account for 0.5-5% of mediastinal tumors (1, 2). We present here a unique case of PMS with paraneoplastic encephalitis. To our knowledge, only six additional cases have been previously described around the world. This is likely due to the rarity of the tumor, but also because this tumor type can be difficult to diagnose. Prior to full histologic evaluation, these tumors are often mistaken for thymic or thyroid neoplasms (3–5). For example, seminoma tumor cells have a CD117+ immunohistochemical (IHC) phenotype, a pattern also seen in thymic tumors (6). Selective IHC stain patterns are emerging and may be able to assist in effective tumor identification (7). In a recent study of PMS published by Fichtner et. al, staining for OCT3/4, D2-40, and TdT along with CD117+ were specific for seminoma histology identification (7). Cellular histology of seminomas demonstrates epithelioid cells with glycogen-rich cytoplasm and nucleoli that resemble primordial germ cells (6). Histology of PMS is identical to that of testicular seminomas.

Despite presenting as a diagnostic challenge, PMS can present with distinct complications often related to mass effect. Known common complications of mediastinal tumors include superior vena cava (SVC) syndrome (8), chylothorax (9), pericardial effusion (10), and lymphoid hyperplasia (5, 11). When planning for surgical removal, these tumors require remarkable precision, skill, and planning due to the nature of their location and adjacency to surrounding tissues. This tumor type’s response to surgery as well as chemotherapy and/or radiation has been shown to be favorable. Even under circumstances where these tumors are invading nearby structures, PMS have been shown to have excellent survival rates (12, 13). Overall survival rates at 5 and 10-year timepoints range from 87% to 100% and from 75% to 100% (14). Rarely, these tumors can present with the aforementioned complications as well as paraneoplastic encephalitis. The specific survival outcomes of patients with PMS and paraneoplastic encephalitis are not well-defined, as these tumors present a complex clinical picture requiring extensive neurologic, oncologic, and often surgical treatments.

## Case presentation

**Figure 1:**
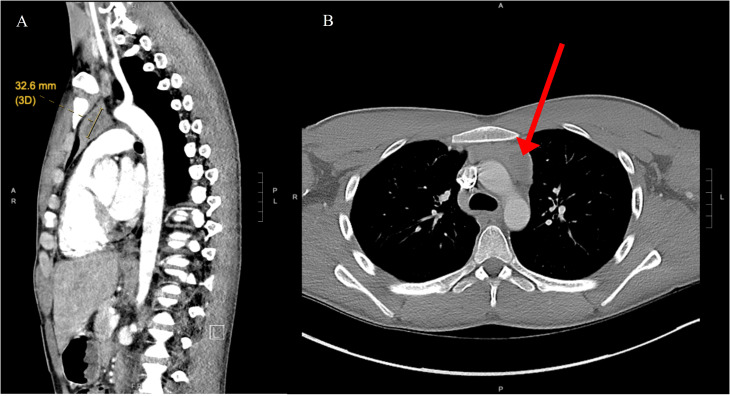
**(A)** Nodular area with central low density in the left side of the thymus, measuring roughly 32 mm long axis indicative of mediastinal seminoma (yellow indicator). **(B)** Computed tomography of axial chest demonstrates anterior mediastinal mass (red arrow).

**Figure 2:**
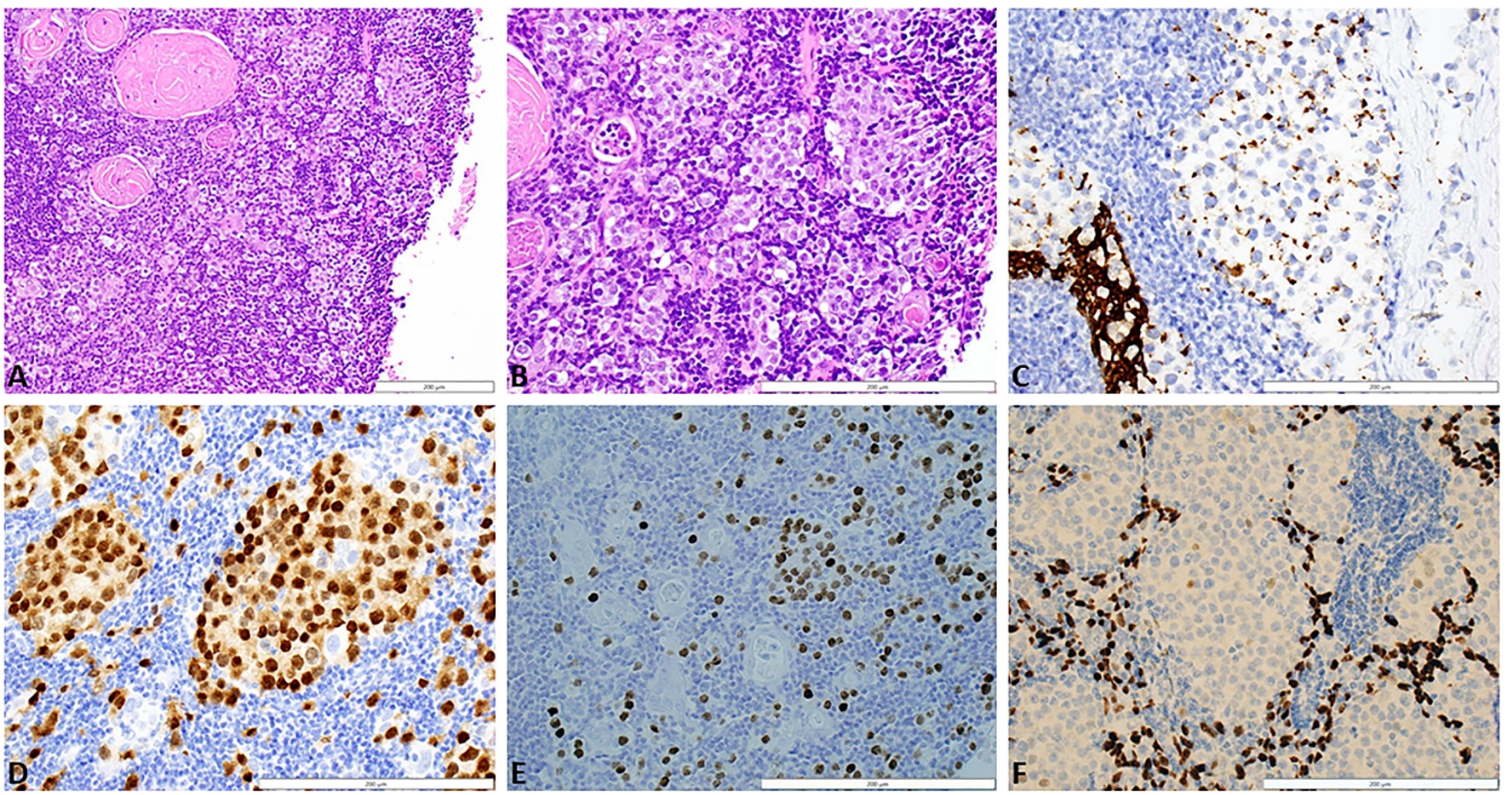
**(A)** Section of thymus shows cystic degeneration at the right side of the image (white space) and a rim of thymus showing intact, normal Hassel's corpuscles and a diffusely infiltrative malignant process. Tumor cells are present as single, dispersed cells, as well as cords and clusters, in a background of lymphocytes and thymic epithelial cells. Although there is cystic degeneration, the diffusely infiltrative growth pattern results in more of thickening of the thymic epithelium rather than a discrete mass. H&E stain, 20x magnification. **(B)** Higher magnification showing tumor infiltrative as single cells, cords, and clusters between lymphocytes, thymic epithelium, and Hassel's corpuscles. The tumor cells are large, contain clear to eosinophilic cytoplasm, and have large, round nuclei with prominent nucleoli. H&E stain, 40x magnification. **(C)** Pancytokeratin stain shows diffuse staining in the normal thymic epithelium in the lower left portion of the image. In contast, clusters of tumor cells show focal, punctuate staining. AE1/AE3 stain, 40x magnification. **(D)** Clusters and isolated seminoma cells show nuclear posibility with the germ cell marker OCT3/4. OCT3/4 stain, 40x magnification. **(E)** Clusters and isolated seminoma cells show nuclear positivity with the germ cell marker SALL4. SALL4 stain, 40x magnification. **(F)** Background thymic epithelium stain positively with p63 while the seminoma cells that are present in clusters are negative. p63 stain, 40x magnification. H&E, Hematoxylin and eosin; AE1/AE3, cytokeratin stain; OCT 3/4, octamer-binding transcription factor 4; SALL4, spalt like transcription factor 4.

**Figure 3:**
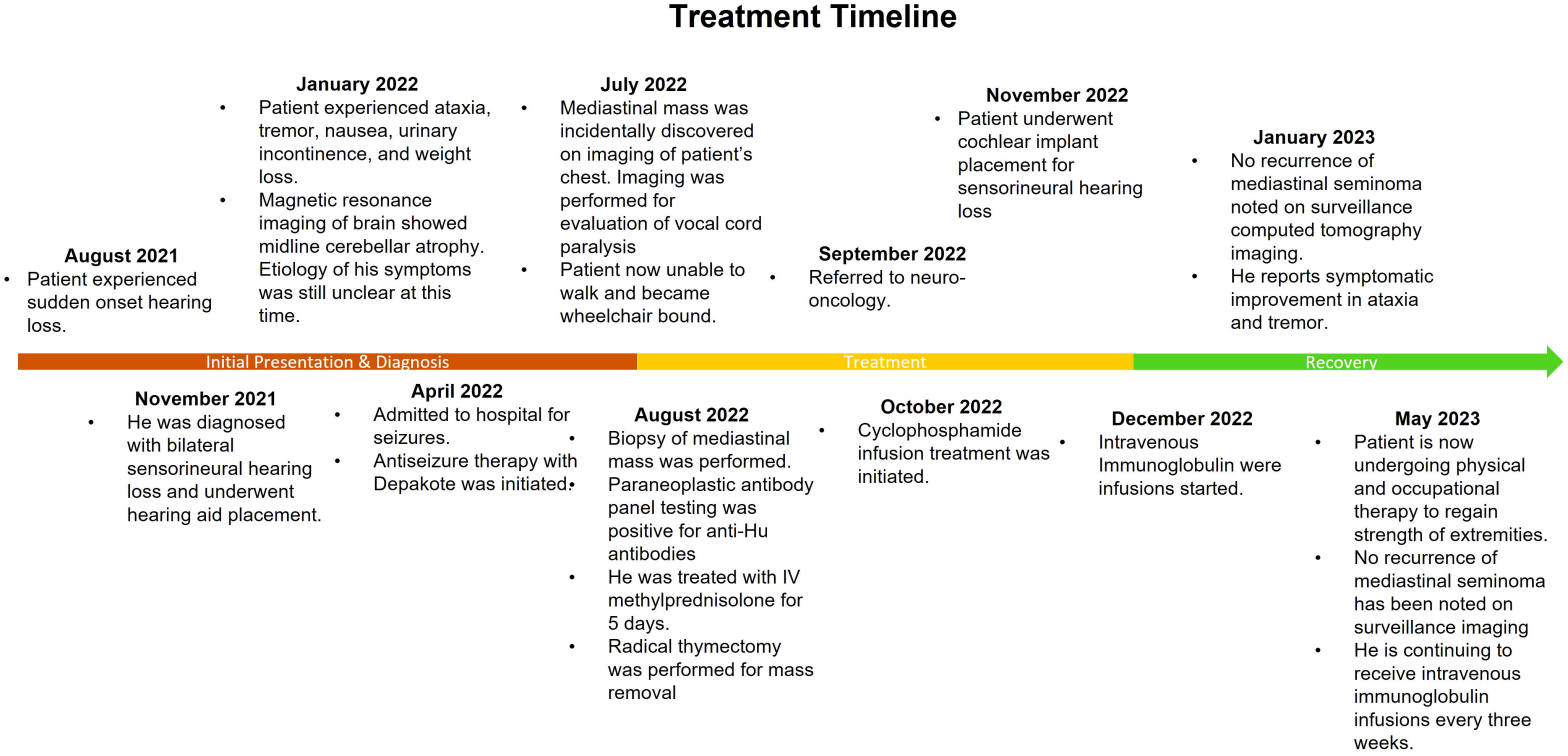
A detailed diagnosis and treatment timeline of mediastinal seminoma with paraneoplastic anti-Hu encephalitis.

A 19-year-old male with no significant past medical history presented with a chief complaint of hearing loss. Initial otolaryngology work-up revealed severe bilateral sensorineural hearing loss, and he was fitted with hearing aids. Shortly thereafter he began to experience progressive dizziness, neuropathy, ataxia, hand tremor, and generalized seizures. He had no family history of testicular cancer. No past medical history of Klinefelter syndrome. Physical exam was significant for symptoms of cerebellar impairment, including wide-based gait with varying stride length, and prolonged finger-to-nose testing. Extensive work up was performed including serum neoplastic antibody panel: ANNA1 (Anti-Hu), ANNA2S (Anti-Ri), ANNA3S, P/Q-type Calcium Channel, CRMP-5-IgG, PCA2, PCA1,PCA-TR, Neuronal Voltage-Gated K+ Channel, AGNA-1, Amphiphysin, MOGFS Myelin Oligodendrocyte Glycoprotein (MOG-IgG1), NMO/AQP4 FACS, and Muscle-Specific Kinase (MuSK) were normal except Anti-Hu (titer: 1:3840); tumor markers: serum beta-hCG, and alpha fetoprotein were normal. LDH also was within normal limits at 196 (normal 116 – 250). No lumbar puncture or CSF studies were performed, as antineoplastic antibodies were detected prior to procedure. Computed tomography (CT) head with contrast was unremarkable. Magnetic resonance imaging (MRI) head performed with and without IV contrast demonstrated midline cerebellar atrophy. No restricted diffusion noted on MRI. No mass lesions noted. Video electroencephalogram performed was normal without evidence of epileptiform activity. CT chest with contrast revealed a heterogeneous anterior mediastinal soft tissue nodule located within the thymus (**Figure 1**). Initial CT-guided core mediastinal mass biopsy demonstrated findings suggestive of thymic neoplasm. Subsequently, the patient underwent median sternotomy with radical thymectomy. Pathology revealed seminoma with lymphoid hyperplasia and cystic change with IHC positive for pancytokeratin, CD117, octomer-binding transcription factor 3/4 (Oct3/4), placental alkaline phosphatase (PLAP), and spalt like transcription factor 4 (SALL4) (**Figure 2**). He was diagnosed with rhombencephalitis, paraneoplastic syndrome secondary to PMS. CT abdomen/pelvis was completed for staging and did not demonstrate any evidence of malignancy or metastatic disease. He was given intravenous (IV) methylprednisolone with mild improvement in his neurological symptoms. He is currently undergoing intravenous immune globulin (IVIG) treatment for his rhombencephalitis. No reported seizures and his neuropathy remains stable. Regarding his cancer, he is under surveillance as his repeat post-operative CT chest did not identify any new suspicious nodules and tumor markers were normal. He works with physical and occupational therapists and is now able to walk with walker (**Figure 3**).

## Discussion

Anti-Hu associated paraneoplastic rhombencephalitis is uncommonly associated with mediastinal germ cell tumors. Also known as antineuronal nuclear antibody (ANNA-1), presence of anti-Hu antibodies can result in varying neurologic disturbances, such as psychoses, seizures, hemiparesis, unexplained fevers, visual disturbance, and hearing loss. As the symptoms of paraneoplastic syndromes that occur secondary to PMS are nonspecific, diagnosis of these tumors can be difficult to ascertain. The mediastinum is also common location for thymomas, thyroid carcinomas, and T-cell lymphoma (4). Thyroid carcinoma and seminoma tissue have similar histologic features, which can result in misdiagnoses. Staining for markers such as SALL4, PLAP, and OCT3/4 are key in the process of clarifying a mediastinal seminoma diagnosis (4, 7).

In addition to our documented case of PMS with paraneoplastic rhombencephalitis, there are six additional reported cases as described in **Table 1**. All of the patients were male with ages ranging from 19 to 49 years old. Similar to our patient, a 45-year-old male in Israel presented with bilateral sensorineural hearing loss (17). He was found to have anti-KLH11 antibodies which have been described previously in Kelch-like protein 11 antibody-associated paraneoplastic neurological (KELCH) syndrome. Anti-KLH11, first described in 2019 as a new marker for paraneoplastic encephalitis, has been postulated as more likely to be associated with germ cell tumors such as seminoma (21). Cases reviewed in a literature review by Li et al. documented that patients with anti-KLH11 also presented with other paraneoplastic antibodies such as anti-leucine zipper 4, anti-Ma2, and anti-Hu (21). In an additional PMS case, a 21-year- male from Germany who presented with panhypopituitarism was found to have Anti-Ma2 antibodies (19). Another case describes a 19-year-old male, also with PMS and Anti-Ma2 antibodies (16). In France, a 49-year-old male presented with signs of panhypopituitarism as well as progressive behavioral disorder, right hemiparesis, and malignant fever. He was found to have PMS with Anti-Ri antibodies (15). A 20-year-old male with PMS, psychosis, and suicide attempts was found to have Anti-SS DNA 189 u/ml antibodies (18). A 32-year-old male from Germany with PMS was found to have concurrent anti-Hu and anti-NMDA receptor antibodies (20).This is the only additional reported case of PMS presenting with anti-Hu paraneoplastic encephalitis.

**Table 1:** In addition to our documented case of paraneoplastic encephalitis with primary mediastinal seminoma there are six additional documented cases.

Publication	Patient Demographics	Country of Publication/Case	Initial Presenting Symptoms	Associated Antibodies	Treatment	Outcome
Launay et.al (15).	49-year-old Male	Hospital Pasteur Nice, France	Progressive behavioral disorder panhypopituitarism, right hemiparesis, left common oculomotor nerve paralysis, fever	anti-Ri	Corticosteroids Carboplatin Hormone replacement therapy	Paraneoplastic fever disappeared, but behavioral disorders and palsy remain unchanged
Bosemani et. al (16).	19-year-old male	Johns Hopkins University School of Medicine United States Baltimore, Maryland	Fatigue, daytime sleepiness, headaches, hallucinations, disorientation	anti-Ma2	Immunotherapy Intravenous steroids Plasmapheresis Intravenous immunoglobulin Rituximab Surgical resection of tumor	At 12 weeks' follow-up, his condition was unchanged
Krivitski et. al (17).	45-year-old male	Tel Aviv Medical Center Israel	Bilateral sensorineural hearing loss, vertigo, progressive ataxia	anti-KLHL11	Surgical resection Chemotherapy (unspecified) Cyclophosphamide	Stabilization of symptoms
Plioplys et. al (18).	20-year-old male	University of Illinois United States Chicago, Illinois	Psychosis with delusions, suicide attempts, pleuritic chest pain	Anti-SS DNA 189 u/ ml	Surgical resection Venlafaxine Risperidone No chemotherapy treatment mentioned	Improvement of mood and enjoyment of life, returned to work, flat affect persisted, inappropriate affect persisted
Bergner et. al (19).	21-year-old male	University of Gottingen Germany	Headache, hypotonia hypothermia, somnolence, panhypopituitarism	Anti-Ma2	Prednisolone No resection or chemotherapy described in report	Not described in report
Pohley et. al (20).	32-year-old male	University of Rostock Rostock, Germany	Headaches, muscle pain weight loss, generalized epileptic seizures, optic and acoustic hallucinations, oculomotor disturbances, gait ataxia, polyneuropathy	Anti-Hu Anti-NMDA receptor	Prednisolone Intravenous immunoglobulin Plasmapheresis	Per report, treatment did not resolve symptoms

Anti-Ri: Anti-neuronal nuclear antibodies type II, also known as ANNA-2.

Anti-KLHL11: Anti-Kelch-like protein 11 antibody.

Anti-SS DNA: Anti-single stranded deoxyribonucleic acid antibody.

Anti-Hu: Type 1 antineuronal nuclear antibody, also known as ANNA-1.

Anti-NMDA receptor: Anti-N-methyl-d-aspartate receptor antibody.

Patients in each of these cases underwent a variety of treatments, including chemotherapy, plasmapheresis, IVIG, and surgical removal. A recently published study performed by Zhai et al. compared outcomes of patients with PMS who underwent surgical treatment versus chemotherapy +/- radiation. This single-center retrospective study in 27 patients showed that there was no significant difference in 5-year overall survival, cancer-specific survival, and progression-free survival between patients with and without surgery (1). In contrast, a population-based study performed in China with 476 patients sought to predict the survival of patients with PMS (22). One factor found to negatively impact overall survival was not undergoing surgery (22).

While outcomes of patients with PMS are well-described in the literature, outcomes of patients with PMS and concurrent paraneoplastic encephalitis are not articulated. The paucity of cases involving PMS with paraneoplastic encephalitis makes it difficult to assess which treatments are the most effective at benefitting the patient’s mortality and improving patients’ symptoms. In reference to the cases discussed here, neurologic recovery and patient outcomes differed on a case-by-case basis in PMS patients with paraneoplastic encephalitis. One case of a 22-year-old male with Anti-SS DNA 189 u/ml antibodies reported a nearly full recovery of cognitive functions with treatment (18), while the patient with anti-KLHL11 reported stabilization of neurologic symptoms (17). It appears that even after surgical removal of the patients’ tumors and immunosuppressive treatment, neurological symptoms persisted in most patients. Our patient was treated with IV and oral prednisone for a short course and is currently undergoing IVIG treatment. His neurological symptoms have mildly improved. He was wheelchair bound and is now able to walk with walker, but his weakness and neuropathy have persisted. The patient with both anti-Hu and anti-NMDA receptor antibodies was treated with a combination of prednisolone, IVIG, and plasmapheresis, however, it was reported that symptoms of generalized seizures, oculomotor and auditory disturbances did not improve (20). The patient with Anti-Ri antibodies underwent tumor resection along with carboplatin, corticosteroids, and hormone replacement therapy. His paraneoplastic fever disappeared, however his abnormal behaviors remained unchanged (15). The 21-year-old male from Germany with Anti-Ma2 was treated with prednisolone reportedly rapidly improved (16). The report did not specify what improvements the patient experienced, or if the patient underwent resection of primary tumor.

In a 2022 published report by Budhram et al, 27 patients with anti-Hu extralimbic encephalitis were identified and patient outcomes were described (23). None of the patients in this study had PMS, however ~80% of the patients did have solid organ malignancy. Many of the patients received chemotherapy and/or immunotherapy as well as immunosuppressive treatment. The majority of patients were placed on steroids. Overall, there was notably good response to treatment with anti-seizure therapy and either steroids or other immunotherapies (23). Most adults (10 out of 19) survived and remained seizure free post-treatment while other adults (8 out of 19) died. Half of the adults who survived and remained seizure-free had persistent neurologic symptoms including dysautonomia, gait ataxia, and hemiparesis.

## Conclusion

In conclusion, PMS is a rare tumor, and presentation with paraneoplastic encephalitis is even more infrequent. A multidisciplinary approach should be considered in the setting of paraneoplastic symptoms. There is a need for more frequent documentation of unique cases in order to better establish treatment approaches that may improve patient outcomes.

## Data availability statement

The original contributions presented in the study are included in the article/supplementary material. Further inquiries can be directed to the corresponding author.

## Ethics statement

Written informed consent was obtained from the individual(s) for the publication of any potentially identifiable images or data included in this article.

## Author contributions

CW: drafting of the article and final approval of the manuscript. ZM: drafting of the article, acquisition of data, and final approval of the manuscript. DA: providing histopathology pictures and final approval of the manuscript. AC and RB: drafting of the article. All authors contributed to the article and approved the submitted version.
